# Childhood and recent stressors: Implications for physical and cognitive health in older adults

**DOI:** 10.1093/geroni/igaf122.2517

**Published:** 2025-12-31

**Authors:** Athena Chan, Sun-Kyung Lee

**Affiliations:** Texas Tech University, Lubbock, Texas, United States; Arizona State University, Phoenix, Arizona, United States

## Abstract

Childhood adversities and recent life stressors are less understood on their joint impact on physical and cognitive health in older adults. This study explores stressor typologies and their association with health. We analyzed data from 14,236 older adults (ages 50-101, M = 67.2) from the Health and Retirement Study (2010-2020). Using a 3-step BCH approach in Mplus, latent class analysis identified profiles based on 10 indicators: childhood adversities (e.g., repeating a grade, trouble with police, parental physical abuse) and recent life events (e.g., natural disaster, life-threatening illness/accident, homelessness). Multinomial logistic regression identified demographic predictors of profiles. Health was predicted after 10 years, adjusting for these predictors. Four latent classes emerged: Low stressors (75%), Childhood physical abuse/substance household (10%), Lifetime law enforcement/adulthood homelessness (5%), and High recent life events (9%). Compared to Low stressors, Childhood physical abuse was more likely to be White and female, while Lifetime law enforcement and High recent life events classes were more likely to be male and ethnic/racial minorities. After adjusting for demographics, no significant class differences were found in dementia risk. However, Childhood physical abuse and Lifetime law enforcement classes had lower cognitive impairment, while Low stressors had the highest. High life events class had the most chronic conditions, and Lifetime law enforcement had the highest lifestyle risk. These findings highlight how cumulative stressors impact health in older adulthood, with certain stressor types linked to cognitive decline and physical health problems. Understanding stressor history may inform tailored health interventions and care strategies for older populations.

